# Enhanced labeling density and whole-cell 3D *d*STORM imaging by repetitive labeling of target proteins

**DOI:** 10.1038/s41598-018-23818-0

**Published:** 2018-04-03

**Authors:** Varun Venkataramani, Markus Kardorff, Frank Herrmannsdörfer, Ralph Wieneke, Alina Klein, Robert Tampé, Mike Heilemann, Thomas Kuner

**Affiliations:** 1 Department of Functional Neuroanatomy, Institute for Anatomy and Cell Biology, Heidelberg University, Im Neuenheimer Feld 307, 69120 Heidelberg, Germany; 2 Institute of Biochemistry, Biocenter, Goethe-University Frankfurt, Max-von-Laue-Str. 9, 60438 Frankfurt/M, Germany; 3 Institute of Physical and Theoretical Chemistry, Goethe-University Frankfurt, Max-von-Laue-Str. 7, 60438 Frankfurt/M, Germany

## Abstract

With continuing advances in the resolving power of super-resolution microscopy, the inefficient labeling of proteins with suitable fluorophores becomes a limiting factor. For example, the low labeling density achieved with antibodies or small molecule tags limits attempts to reveal local protein nano-architecture of cellular compartments. On the other hand, high laser intensities cause photobleaching within and nearby an imaged region, thereby further reducing labeling density and impairing multi-plane whole-cell 3D super-resolution imaging. Here, we show that both labeling density and photobleaching can be addressed by repetitive application of trisNTA-fluorophore conjugates reversibly binding to a histidine-tagged protein by a novel approach called single-epitope repetitive imaging (SERI). For single-plane super-resolution microscopy, we demonstrate that, after multiple rounds of labeling and imaging, the signal density is increased. Using the same approach of repetitive imaging, washing and re-labeling, we demonstrate whole-cell 3D super-resolution imaging compensated for photobleaching above or below the imaging plane. This proof-of-principle study demonstrates that repetitive labeling of histidine-tagged proteins provides a versatile solution to break the ‘labeling barrier’ and to bypass photobleaching in multi-plane, whole-cell 3D experiments.

## Introduction

Optical imaging beyond the diffraction limit has become part of the standard repertoire in cell biology. Super-resolution microscopy is suitable to visualize the spatial arrangement of proteins with near-molecular resolution and to quantify the composition of protein complexes^1^. Crucial for all advanced super-resolution microscopy experiments are robust and ideally stoichiometric methods to label protein targets with fluorescent reporters that achieve a high labeling efficiency. While this seems to be given when fusing the protein of interest with a fluorescent protein, either in a knockout background or even better as a knock-in, the resolution is limited by the smaller number of photons typically emitted by fluorescent proteins until they photobleach. Furthermore, not every fluorescent fusion protein may mature into a photon-emitting state, potentially yielding labeled fusion proteins invisible in the experiment. The most commonly applied alternative strategy is labeling with small organic fluorophores, either by immunofluorescence, by introducing chemical tags, or by fluorophore-labeled ligands such as toxins that bind to a specific target. While the photon-yield of organic fluorophores is larger than that of fluorescent proteins and hence the achievable localization precision is smaller, some labeling strategies suffer from low efficiencies and unspecific binding, hampering the structural resolution^2^. Average labeling densities of fluorophore-conjugated antibodies are in the range of 10–20%. Enzyme tags, such as the SNAP-tag, can reach a labeling efficiency of around 50%^3^. An important reason for this observation may arise from the size of labeling probes such as antibodies limiting access to their binding sites, especially in regions of dense protein arrangements. Small probes can bypass this size limitation, e.g. the “small labeling pair” (SLAP) tag that builds on the specific interaction between a short peptide sequence, His_6_ or His_10_, and the trivalent *tris-*N-nitrilotriacetic acid (*tris*NTA)-fluorophore conjugate. The suitability of the SLAP-tag for single-molecule^4^ and super-resolution microscopy in both fixed^5^ and live cells^6,7^ was recently demonstrated using direct stochastic optical reconstruction microscopy (*d*STORM)^8^.

The labeling efficiency can also be increased by reversible binding of fluorophore-labeled probes that are constantly supplied with the imaging buffer to a target molecule, as used in points accumulation for imaging in nanoscale topography (PAINT)^9^, universal PAINT (uPAINT)^10^, and super-resolution by power-dependent active intermittency and PAINT (SPRAIPAINT)^11^. A variant of these methods is DNA-PAINT^12^, which builds on repetitive and reversible binding of fluorophore-labeled imager-strands of single stranded DNA to a complementary target strand, which in case of protein labeling is tagged to a protein-specific antibody, nanobody or small molecule such as phalloidin^13^. DNA-PAINT is particularly powerful for multiplexing applications, achieved by the large number of specific oligonucleotide pairings that can be generated^14^. Applying sequential rounds of supplying specific imager strands, imaging and washing, the same fluorophore can be used for multiple targets, thereby avoiding chromatic aberration and allowing the use of less complex optical setups. The toolbox of DNA-PAINT methods was extended by exchange-*d*STORM, a sequential experimental procedure that first supplies fluorophore-labeled imager strands that stably bind to a specific target, followed by *d*STORM imaging and washing-off of the imager strands by buffer exchange^15^. Exchange-*d*STORM allows for sequential multiplexed imaging with the same fluorophore. Another recent addition is FRET-PAINT^16^, which efficiently reduces the background signal generated by DNA imager strands in solution. Consequently, FRET-PAINT allows for widefield and three-dimensional imaging. Apart from DNA oligonucleotides, also protein fragments that directly associate and dissociate from their target structures enable PAINT-like super-resolution imaging^17,18^.

Here, we introduce an experimental protocol for single-epitope repetitive imaging (SERI) that builds on multiple rounds of labeling with a small labeling pair, *tris*NTA-dye and His_10_-tagged proteins, and on the fact that the non-covalent interaction between the two binding partners is in competition to imidazole added with a washing buffer^5^. Through multiple rounds of labeling, *d*STORM imaging and elution, we demonstrate super-resolution microscopy with increased signal density. This approach is in particular useful for volume imaging as it re-compensates photobleaching of fluorophores by replenishing fresh labels in each round of imaging.

## Results

**Figure 1:**
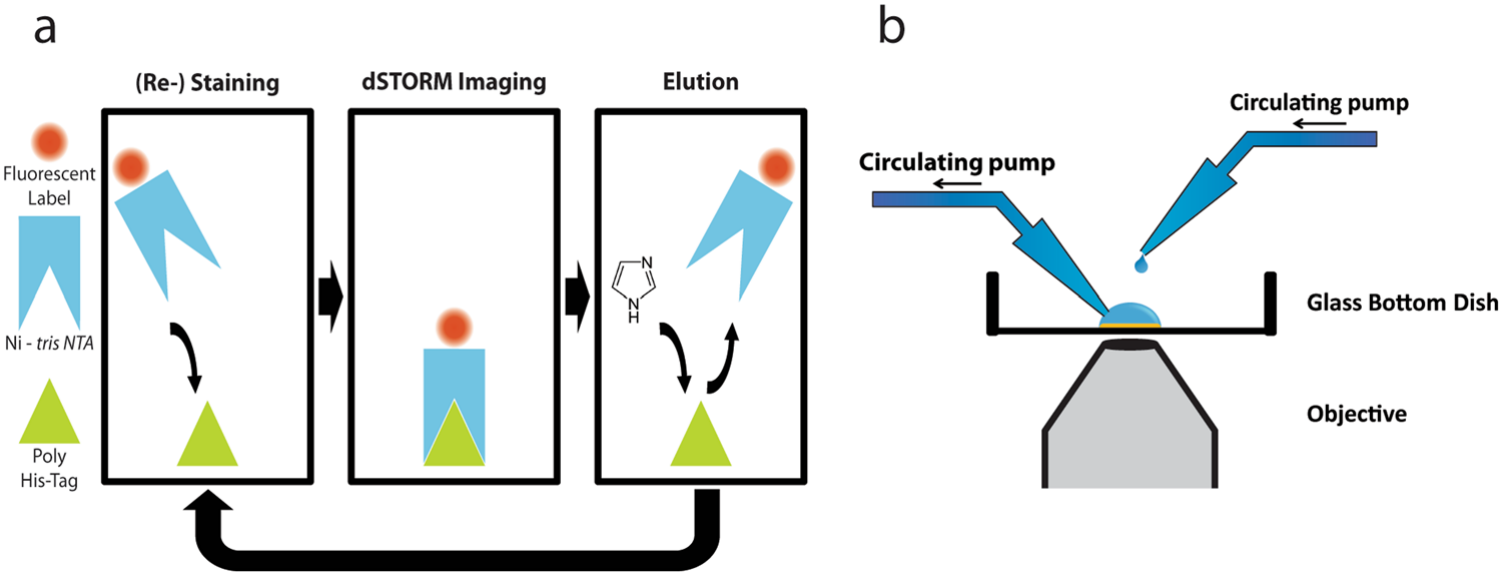
The principle of SERI and its technical implementation. (**a**) Fluorophore-labeled *tris*NTA binds to His_6_/His_10_-tagged proteins and enables *d*STORM super-resolution imaging. *tris*NTA can be eluted using a buffer containing imidazole. This procedure of labeling, imaging and elution can be repeated many times, with each repetition providing a super-resolution image. (**b**) Schematic depiction of the restaining setup. Two circulating pumps with plastic tubing allow for solution exchange during washing steps.

We designed an experimental protocol consisting of multiple sequences of labeling, imaging, and elution of *tris*NTA-fluorophores targeting a His_10_-tagged protein (Fig. 1a). The nanomolar (nM) affinity interaction of the His_10_-tag with *tris*NTA allows for specific binding and an off-rate sufficiently slow for *d*STORM imaging. Yet, washing the sample with a buffer containing 150 mM imidazole reliably removes the fluorophore label from the target without harming cellular structures^5^. Hence, many cycles of labeling, imaging, elution, and re-labeling can be achieved. We implemented this configuration on an inverse microscope with two circulating pumps for semi-automatic fluid exchange (Fig. 1b).

**Figure 2:**
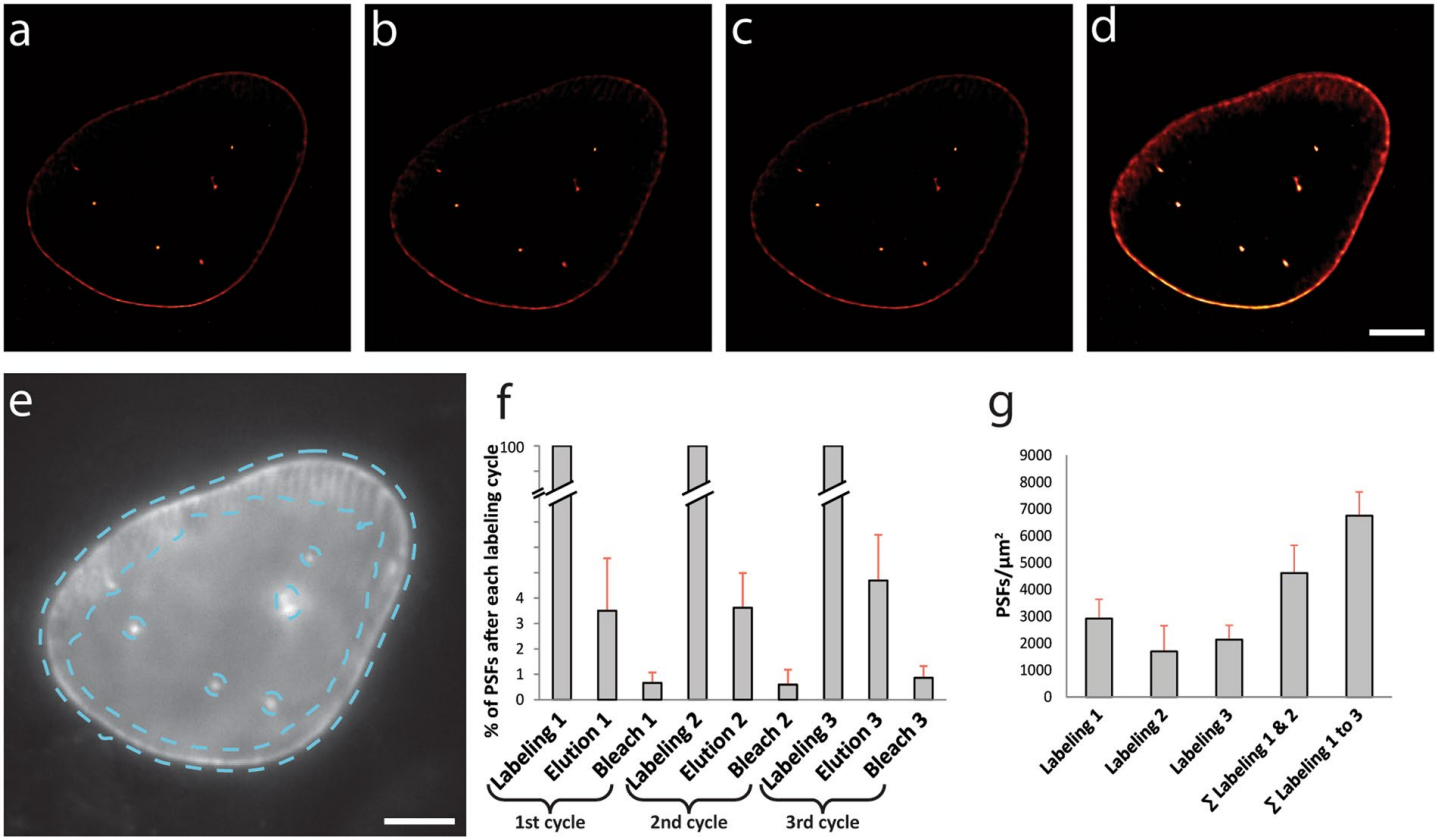
Contrast-enhanced super-resolution imaging of His_10_-mEGFP-LaminA with SERI. (**a**–**c**) Representative super-resolution images obtained in repetitive rounds of *d*STORM imaging of His_10_-mEGFP-LaminA expressing U2OS cells. (**d**) Sum image generated from the three labeling and imaging rounds. (**e**) Widefield image showing the mask (cyan-colored dashed line) used for quantification of single-molecule detection events. The mask was created by smoothing and thresholding the overlaid *d*STORM images. (**f**) Quantification of elution and bleaching. The relative number of single-molecule localizations (as point-spread functions, PSF) on the laminA structure was quantified. All PSFs of each elution and bleaching cycles were normalized to the respective bleaching-corrected labeling round. (**g**) Each labeling cycle and the summations of labeling densities over three labeling rounds (bars in (**f**,**g**) represent mean ± SD, N = 6 independent cells) (scale bars: 5 µm).

To demonstrate the SERI concept, we expressed His_10_-mEGFP-LaminA in U2OS cells, stained the cells with *tris*NTA-AlexaFluor647 and recorded *d*STORM images of single cells. The images shown in Fig. 2 reflect the preferred localization of lamins underneath the nuclear membrane (near continuous line with infoldings) and at distinct nucleoplasmic sites (dots within the nucleus). Following imaging, we eluted the fluorophore probe from the sample using an imidazole-containing washing buffer (see Methods for a detailed description), re-labeled the sample with *tris*NTA-AlexaFluor647, and recorded another *d*STORM image (Fig. 2a–c). The repeated cycles of labeling, imaging, and elution generated multiple super-resolution images that can be merged into one image thereby achieving an increased signal density (Fig. 2d). To demonstrate super-resolution of  these images, we determined the localization precision from each single staining round using the nearest-neighbor method^19^. We found an average localization precision of 24.1 ± 4.6 nm (mean ± SD), consistent with super-resolved images (a higher magnification image is not shown here because the lamin network is too dense to be resolved with this resolution, see^20^).

While this increase in signal density may reflect a higher degree of labeling by *tris*NTA-AlexaFluor647 binding to new target molecules in each round of labeling, further experiments are required to support such a mechanism. At this stage of a proof of concept, we point out at a clear increase in image contrast that is observed with SERI and is practically useful (compare Fig. 2a–c to Fig. 2d).

We next quantified the reliability of SERI by determining the number of single-molecule localizations per area in each round of imaging. We recorded *d*STORM images of a U2OS cell expressing His_10_-mEGFP-LaminA labeled with *tris*NTA-AlexaFluor647 before and after elution with imidazole, followed by illumination with high laser intensities to induce photo-bleaching of the remaining fluorophores (Fig. 2e). Elution with imidazole reduced the number of localizations to below 4% and additional photobleaching to even less than 1% of the original number of localizations in the labeled sample (Fig. 2f). The fraction of unbleached fluorophores after recording of the first 11,000 frames was estimated by using a bleaching model (see materials and methods). The number of single-molecule localizations for consecutive rounds of *d*STORM imaging were similar for each round, yet accumulated with each round to yield a much higher total number of localizations (Fig. 2g).

For all re-staining experiments, we implemented a simple approach for liquid handling on our microscopy setup, thereby minimizing efforts of aligning image frames due to displacements of the sample. The stage coordinates were saved for each position and up to ten different positions per staining cycle were imaged. All cells could be re-identified and registered over different staining cycles. Each single round of imaging produced super-resolved images of LaminA that are well comparable to previously published super-resolved images of this structure using dSTORM imaging and the same small labeling pair^5,7,21^. Additional rounds of staining were performed in a time-efficient way with elution for 5 min and re-staining with *tris*NTA-AlexaFluor647 for one hour. We provide proof of principle that SERI increases the signal density and thus image contrast when adding up localizations of all imaging rounds. After three rounds of labeling, imaging and elution, the cumulated signal intensity is approximately three times higher than in single experiments and has not yet reached saturation (Fig. 2f,g). In addition, we determined the signal-to-noise ratio of single super-resolution images and compared it to the cumulated image. We found that restaining and reimaging of the samples significantly increase the signal-to-noise ratio (see Methods, Supplementary Figure 1).

**Figure 3:**
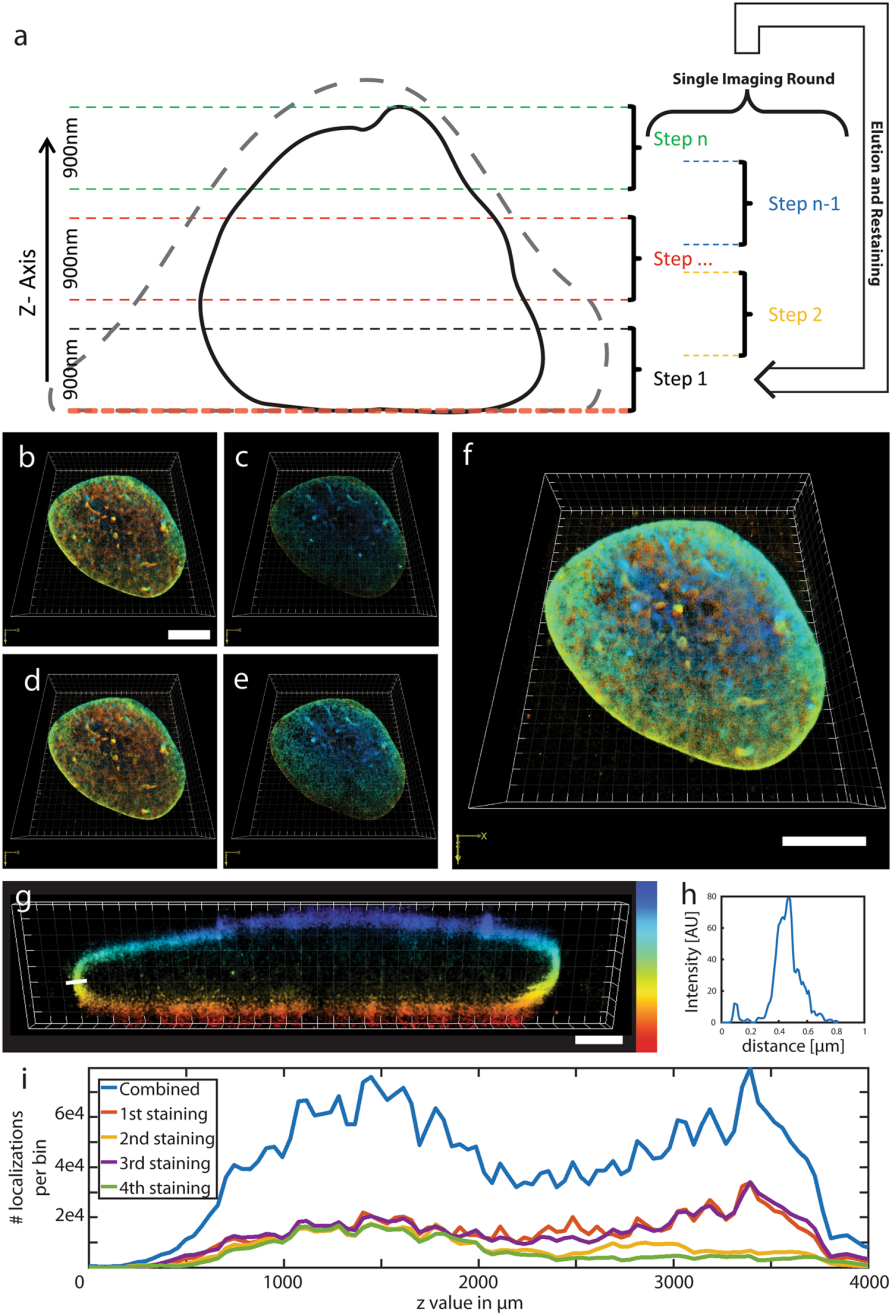
3D large volume imaging of laminA-His_10_-eGFP. (**a**) Schematic depiction of imaging an entire cell (stippled line), here mostly represented by the nucleus (continuous line), with SERI. The focus is shifted after a defined number of frames during a single imaging cycle to cover the entire volume. Thereafter the cells are treated with elution buffer, re-stained and imaged again. The stippled line denotes the assumed contours of the cell. (**b**–**e**) Four different single imaging rounds of a cell transfected with His10-mEGFP-LaminA. Note that measurements 1 (**b**) and 3 (**d**) were done in top-down direction while measurements 2 (**c**) and 4 (**e**) were done in reverse direction, thereby revealing the bleaching-induced decrease of the number of localizations in the direction of image acquisition (indicated by color code for z position). (**f**) Overlay of all four single imaging rounds. The scale bars in (**b**,**f**) correspond to 5 µm of the bottom side of the cell. The scale bar in (**b**) applies to panels (**b**–**e**). (**g**) xz-projection of a 500 nm thick section. The color bar ranges from 0 µm (red) to 4 µm (blue). (**h**) Intensity cross section of the lamin border. (**i**) Distribution of localizations over the z-positions of the nucleus of each measurement and of the merged measurements. The merged measurement demonstrates an enhanced labeling density over the z-range in comparison to single measurements. Similar results were obtained in a total of N = 4 cells.

Another challenge in super-resolution microscopy is large volume imaging, which often suffers from photobleaching of fluorophores above or below the actual imaging plane^2,22^. This challenge can be addressed with fluorescent probes that specifically target cellular structures as illustrated by imaging multi-cellular samples with fluorogenic PAINT-probes^2^. Here, we explored how the SERI concept can provide a labeling density sufficient for 3D super-resolution imaging of volumes as large as whole-cells. We chose U2OS cells expressing His_10_-mEGFP-LaminA to demonstrate whole-cell imaging, since the protein LaminA covers the whole nucleus and represents a large volume structure within these cells. After *tris*NTA-AlexaFluor647 staining, entire nuclei of U2OS cells expressing His_10_-mEGFP-LaminA were imaged in 11 mutually overlapping slabs of approximately 900 nm thickness using astigmatism-based 3D *d*STORM (Fig. 3a). This was achieved by using a piezo-based objective positioner calibrated to move the objective by approximately 550 nm during each imaging step. Thereafter, *tris*NTA-AlexaFluor647 was eluted with imidazole buffer and three more rounds of staining, imaging of 11 consecutive slabs in reverse order and elution were performed (Fig. 3b–e). Similar to single-plane imaging shown above, the labeling density increased continuously over several imaging rounds as evident in the cumulated image (Fig. 3f). The images show that laminA forms a continuous layer of about 200 nm thickness underneath the nuclear membrane (Fig. 3g,h). The pronounced bleaching of fluorophores situated, depending on the direction of stack acquisition, above or below the imaging plane, is evident in the z-intensity profiles of the individual measurements (Fig. 3i). Merging the individual imaging rounds strongly improved the labeling density in the z dimension (Fig. 3i). In conclusion, photobleaching of neighboring imaging slabs was compensated for by repetitive replenishment of the *tris*NTA-AlexaFluor647, thereby demonstrating the feasibility of large volume 3D super-resolution imaging at the scale of entire cells.

## Discussion

We introduce a novel combined repetitive labeling and imaging approach with a small labeling pair of *tris*NTA-Alexa-Fluor647 and oligohistidine-tagged proteins. Our approach allows reversible targeting of proteins similar to DNA-PAINT, but does not require the use of antibodies. Antibody labeling suffers from a low labeling efficiency and a large distance between label and respective epitope of approximately 7.5 nm^23^. In comparison, the distance between oligohistidine-tags and *tris*NTA-AlexaFluor647 is in the range of 1 nm, which places the fluorophore very close to the target protein^5,23^. This is especially favorable as the localization precision constantly improves to the point of near-molecular resolution^1^.

We chose His_10_-mEGFP-LaminA for proof-of-principle experiments, because it easily allows to differentiate between background labeling (mostly within the nucleus) and labeling of the true structure that encompasses the nuclear envelope. In addition, LaminA staining allows imaging a large volume structure in U2OS cells, which demonstrates the capability of our method. We quantified the efficiency of SERI in multiple rounds of elution, bleaching, and re-staining: (i) the elution protocol reliably elutes more than 95% of the original labels; (ii) elution does not disrupt the underlying structure, because each imaging cycle could be reliably superimposed; (iii) labeling density increased with each round of labeling and imaging, yielding a more detailed image (Fig. 2g); (iv) SERI enables whole-cell super-resolution microscopy.

In principle, any other cellular protein could be imaged with the SERI approach and also other suitable labeling pairs could be used. It may depend on individual features of the protein such as abundance and spatial arrangement that will define successful application of SERI. Furthermore, small organic labels may produce signal background in SMLM experiments because of unspecific binding of the *tris*NTA-fluorophore conjugates. Experimental protocols that reduce unspecific binding, e.g. by adding low concentrations of imidazole, and the properties of various fluorophores have been explored previously^5,7^.

Other approaches employing repetitive labeling with multiple target structures have been reported before. Tam *et al*. report chemical bleaching with sodium borohydride treatment of samples after imaging allows for multiplexed imaging^24^. The authors compared labeling densities after imaging and found them to be in the same range as the background labeling density. Compared with SERI, this approach utilizes treatments that may interfere with the structural integrity of the sample. Valley *et al*. performed multi-color imaging by sequential labeling, chemical bleaching, photodestruction and relabeling of different cellular targets^25^. The authors report that following photodestruction, only 0.18% of the originally present fluorophores are active. However, this labeling protocol was so far used for multiplexing, but could in principle also be used for repetitive labeling of a same structure. Elution and restaining with antibodies was also introduced for multi-target super-resolution imaging^26^, and could equally be used with the SERI approach. PAINT and PAINT-like approaches also allow for high labeling densities, for imaging of large three-dimensional volumes as well as of multiple targets^14,16,17,22^. The recently introduced FRET-PAINT is a powerful imaging approach that allows for low-background imaging with high labeling densities and similar three-dimensional imaging capabilities as SERI.

In conclusion, we introduce a small labeling pair that can be used for super-resolution imaging with increased image contrast and for whole-cell 3D imaging. We believe that the development of novel labels that allow specific binding and controlled elution will be beneficial, and could further improve and extend this imaging approach.

## Materials and Methods

### Cell Culture

U2OS cells (CLS Cell Line Services, Eppelheim, Germany) were maintained in DMEM (Gibco) with 4.5 g/L glucose (Gibco), supplied with 10% FCS (Gibco), and stored in a humidified tissue culture incubator at 37 °C and 5% CO_2_ atmosphere. Passaging was carried out every 2–3 days using PBS (Sigma-Aldrich) and 0.5% trypsin/PBS (GE Healthcare). Transfection was performed using 18 mM of branched PEI with a DNA/PEI ratio of 1:3, following the manufacturer’s instructions. Cells were seeded into a glass-bottom dish with a number of 2 × 10^4^ cells per well and subsequently transfected with 0.2 µg plasmid DNA His_10_-mEGFP-LaminA per well^5^. After 48 h, cells were chemically fixed with 4% PFA for 30 min.

### Staining His_10_-mEGFP-LaminA with trisNTA-AlexaFluor647

After 24 to 48 h, transfected U2OS cells grown on glass bottom dishes were chemically fixedwith 4% formaldehyde in PBS for 30 min at room temperature. Cells were permeabilized with 0.1% (v/v) Triton X-100 (Sigma-Aldrich) in PBS for 20 min. Cells were washed three times with PBS and incubated with Image-iT FX signal enhancer (Life Technologies) for 30 min at RT. After blocking with 10% BSA in PBS for 60 min, cells were stained with 100 nM trisNTA-AlexaFluor647 in 10% BSA for 1 h followed by washing briefly three times with 10% BSA and three times with PBS.

### 3D *d*STORM imaging

*d*STORM was performed on a custom-built single-molecule microscope configured as described previously^20^. Briefly, for excitation, a laser emitting 660 nm (Coherent Cube) was focused onto the back focal plane of a 100×/1.49-NA (numerical aperture) through-the-objective total internal reflection fluorescence objective (Olympus, Japan). We used a piezo-based objective positioner (Physik-Instrumente, Karlsruhe, Germany) to stabilize the *z*-focus. An electronic feedback loop consisting of an infrared laser beam reflected at the coverslip and a quadrant photodiode tracking the beam position was used to continuously correct the *z*-focus plane. A laser intensity of 2 kW/cm^2^ was used to induce photoswitching of the fluorophores. We used a 405-nm laser to compensate for the consecutively increasing number of bleached fluorophores by increasing the transition rate between the dark state and the emitting state. 33,000 imaging frames were recorded for stained samples using an integration time of 30 ms per imaging round. For all samples, 100 mM freshly prepared MEA in PBS (pH 7.4) was used as imaging buffer. All images were recorded in 3D astigmatism mode. Images presented in Fig. 2 are presented as 2D images to simplify intensity analysis.

### Repetitive super-resolution imaging

Repetitive imaging was performed on His10-mEGFP-LaminA transfected cells stained with *tris*NTA-AlexaFluor647. For quantification, photobleaching and re-staining only 11,000 frames were imaged per imaging round. We chose 11,000 frames to demonstrate in one series of an experiment how well the elution of trisNTA-dyes with imidazole and the restaining works. This required a certain fraction of active fluorophores that are not photobleached at the end of a single imaging round. The number of fluorophores that bleached during the first 11,000 frames was estimated using a model of exponential decrease. The bleach constant was determined from an experiment with similar experimental conditions but with 44,000 frames recorded. After the recording of 11,000 frames, a surviving fraction of approx. 80% was determined. A custom-built semi-automated pipetting setup was used for all solution exchanges. After imaging, samples were briefly washed five times with PBS. *Tris*NTA-AlexaFluor647 were eluted with 250 mM of imidazole for 5 min. For quantification, the samples were photobleached using a 660 nm laser at an intensity of 2 kW/cm^2^ and a 405 nm laser with an intensity of 0.7 kW/cm^2^ for 5 min^25^. Thereafter, samples were restained with 100 nM of *tris*NTA-AlexaFluor647 in PBS supplemented with 10% (w/v) BSA for 60 min and a consecutive imaging round was performed. A total of six cells was imaged and analyzed.

We implemented a configuration of two circulating pumps (Cole Parmer, US) onto our microscope stage: the first pump adds a solution to the glass dishes, and the second pump removes the solution (see Fig. 1b).

For whole-cell SERI a piezo-based objective positioner (Physik-Instrumente, Karlsruhe, Germany) was used to stabilize the z-focus. The objective positioner was calibrated such that the focus could be shifted stepwise (10 times) by approximately 550 nm every 4,000 frames to cover a whole cell volume in a single imaging cycle. Four imaging rounds were performed in four different cells.

For registration of single-plane imaging cycles the Fiji “Template Matching” plugin was used^27^. Whole-cell imaging stacks were registered with a combination of the Template Matching plugin and a custom-written Matlab script.

The signal-to-noise ratio of the images in Fig. 2 was determined as the peak intensity value of the line profile through the LaminA membrane divided by the standard deviation of the background. The results are shown in Supplemental Fig. 1.

### Data availability statement

The datasets generated during and/or analyzed during the current study are available from the corresponding author on reasonable request.

## Electronic supplementary material

supplementary material
