# Supplementary material for: Enhanced labeling density and whole-cell 3D dSTORM imaging by repetitive labeling of target proteins
Source: Sci Rep. 2018 Apr 3;8:5507. doi: 10.1038/s41598-018-23818-0 (PMC5882651; doi:10.1038/s41598-018-23818-0)
Supplement: Supplementary file 1 — supplementary material [file 41598_2018_23818_MOESM1_ESM.docx]

Enhanced labeling density and whole-cell 3D *d*STORM imaging by repetitive labeling of target proteins

Varun Venkataramani^1,+^, Markus Kardorff^1,+^, Frank Herrmannsdörfer^1^, Ralph Wieneke^2^, Alina Klein^2^, Robert Tampé^2^, Mike Heilemann^1,3,*^ and Thomas Kuner^1,*^

^1^ Department of Functional Neuroanatomy, Institute for Anatomy and Cell Biology, Heidelberg University, Im Neuenheimer Feld 307, 69120, Heidelberg, Germany.

^2^ Institute of Biochemistry, Biocenter, Goethe-University Frankfurt, Max-von-Laue-Str. 9, 60438 Frankfurt/M., Germany.

^3^ Institute of Physical and Theoretical Chemistry, Goethe-University Frankfurt, Max-von-Laue-Str. 7, 60438 Frankfurt/M., Germany.

^*^[heilemann@chemie.uni-frankfurt.de](mailto:heilemann@chemie.uni-frankfurt.de), [kuner@uni-heidelberg.de](mailto:kuner@uni-heidelberg.de)

**Supplemental Material**

^
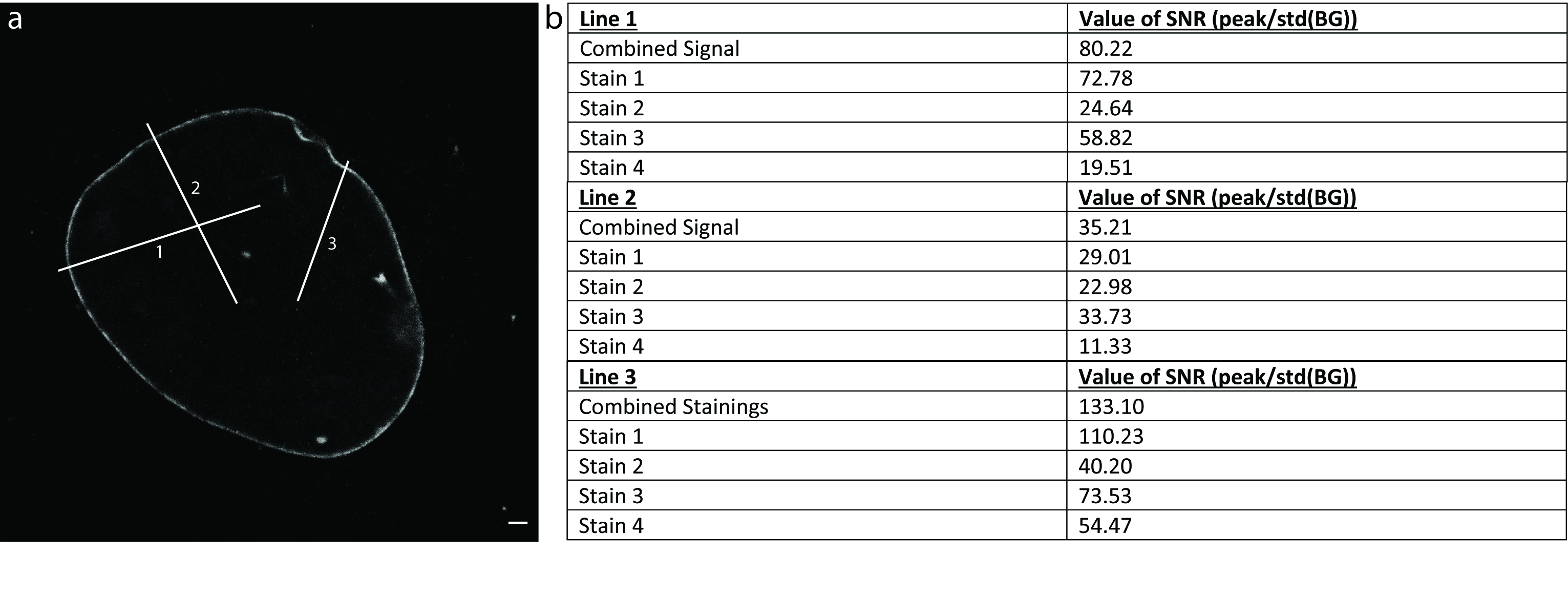
^

**Supplementary Figure 1. Comparison of signal-to-noise ratios from line profiles through the LaminA membrane of single against combined rounds of staining.** (a) 3D-*d*STORM super-position of an experiment with 4 staining rounds of repetitive staining with trisNTA-Alexa647 against His-tagged LaminA. The signal-to-noise ratios of single and combined staining rounds were compared by drawing three randomly chosen lines through the LaminA membrane (scale bar 1 µm). (b) Signal-to-noise ratios of single vs. combined staining rounds. The combined signal-to-noise ratio was always higher than the value determined for a single staining round. The signal-to-noise ratio was determined as the peak intensity value of the line profile through the LaminA membrane divided by the standard deviation of the background.
